# Sentinel Lymph Node Assessment in Endometrial Intraepithelial Neoplasia/Atypical Endometrial Hyperplasia: An Updated Systematic Review and Meta-Analysis

**DOI:** 10.3390/diagnostics16193114

**Published:** 2026-09-25

**Authors:** Pei-Chen Li, Dah-Ching Ding

**Affiliations:** 1 Department of Obstetrics and Gynecology, Hualien Tzu Chi Hospital, Buddhist Tzu Chi Medical Foundation, Hualien 970, Taiwan; ppchen24@gmail.com; 2 Department of Obstetrics and Gynecology, Tainan Sin-Lau Hospital, Tainan 701, Taiwan; 3 Institute of Medical Sciences, Tzu Chi University, Hualien 970, Taiwan

**Keywords:** endometrial intraepithelial neoplasia, atypical endometrial hyperplasia, sentinel lymph node, concurrent endometrial carcinoma, meta-analysis

## Abstract

**Background/Objectives:** Endometrial intraepithelial neoplasia (EIN)/atypical endometrial hyperplasia (AEH) carries a substantial risk of concurrent endometrial carcinoma; however, the role of sentinel lymph node (SLN) assessment during hysterectomy remains controversial. We performed an updated systematic review and meta-analysis to quantify concurrent carcinoma and evaluate SLN/nodal outcomes in women with preoperative EIN/AEH. **Methods:** PubMed, Embase, and the Cochrane Central Register of Controlled Trials were searched for studies reporting concurrent carcinoma and/or SLN/nodal outcomes in women with preoperative EIN/AEH undergoing hysterectomy. Proportions were pooled using a random-effects DerSimonian–Laird model with Freeman–Tukey double-arcsine transformation, with subgroup analyses by histological terminology. Risk of bias was assessed using the QUIPS tool. **Results:** Nineteen studies, including 13,553 women, were included; 14 studies comprising 1174 women contributed to the pooled nodal-positivity estimate. The pooled rate of concurrent carcinoma was 39.6% (95% CI 34.9–44.3; I^2^ = 86%), with rates of 43.8% in AEH/atypical hyperplasia cohorts and 33.4% in EIN cohorts. Pooled nodal positivity was 3.0% (95% CI 2.1–4.0; I^2^ = 0%) and was predominantly low-volume disease. The pooled bilateral SLN detection rate was 71.6% (95% CI 67.3–75.7), and SLN was not associated with excess surgical complications. SLN findings influenced adjuvant treatment decisions in a minority of patients. **Conclusion:** Concurrent carcinoma occurs in approximately two in five women with preoperative EIN/AEH, whereas nodal metastasis is uncommon and generally low-volume. SLN mapping appears feasible and may inform adjuvant management in selected patients. However, because this estimate was derived largely from patients already selected for higher-risk features, it does not, by itself, establish that risk-based selection would substantially outperform routine assessment. Taken together with preliminary prospective data, it is more appropriately viewed as hypothesis-generating support for a selective, risk-stratified approach, pending larger prospective validation.

## 1. Introduction

Endometrial intraepithelial neoplasia (EIN)/atypical endometrial hyperplasia (AEH) is a precursor lesion of endometrioid endometrial carcinoma [1]. Because hysterectomy is the definitive treatment for EIN/AEH and a substantial proportion of patients have concurrent invasive carcinoma at the time of surgery, gynecologic oncologists increasingly consider concurrent surgical staging [2,3]. Sentinel lymph node (SLN) mapping is an established approach to nodal staging in apparent early-stage endometrial cancer and provides accurate nodal assessment with lower morbidity than systematic lymphadenectomy [4]. SLN mapping generally cannot be performed after hysterectomy, requiring the decision to be made preoperatively [5].

Therefore, the clinical dilemma is whether to perform SLN mapping during hysterectomy for EIN/AEH [6], accepting the additional operative time and potential morbidity in the majority of patients who will not have invasive cancer to obtain nodal staging information for the minority who do. Most current guidelines do not recommend routine nodal assessment for EIN/AEH; however, the use of SLN mapping has nevertheless increased substantially in clinical practice [7]. A 2024 meta-analysis of seven studies reported a positive-node rate of only 1.1%; however, subsequent correspondence questioned aspects of its interpretation, and additional primary cohorts have since been published [8].

Against this evolving evidence base, we performed an updated systematic review and meta-analysis to quantify (1) the rate of concurrent endometrial carcinoma, (2) the SLN/nodal-positivity rate, and (3) SLN detection rates, perioperative complications, and the influence of SLN findings on adjuvant treatment decisions in women with a preoperative diagnosis of EIN/AEH. Overall, the updated evidence indicates that concurrent carcinoma is common whereas nodal metastasis is uncommon, supporting consideration of a selective rather than routine approach to nodal assessment.

## 2. Materials and Methods

### 2.1. Protocol and Search

The review followed PRISMA 2020 and was registered with the Open Science Framework (registration; DOI: 10.17605/OSF.IO/VSP8M). PubMed, Embase (Embase.com), and the Cochrane Central Register of Controlled Trials (CENTRAL) were searched from inception to 9 July 2026. The search strategy combined two concept blocks using the Boolean operator AND: (1) EIN/atypical (endometrial) hyperplasia, complex atypical hyperplasia, or endometrial hyperplasia and (2) sentinel lymph node, sentinel node, lymph node mapping, nodal assessment, lymphadenectomy, lymph node dissection, or biopsy. Controlled vocabulary (MeSH/Emtree) was combined with title/abstract terms. No restrictions were applied by language, publication date, or study design (e.g., human-only filters) during the search. Full database-specific strategies are provided in Supplementary File S2. The reference lists of the two 2024 reviews [6,9] and the included studies were also manually searched. Ethical approval was not required because this systematic review analyzed data from previously published studies.

### 2.2. Eligibility

We included primary cohort studies or case series of women with a preoperative diagnosis of EIN/AEH who underwent hysterectomy and reported concurrent endometrial carcinoma on final pathology and/or SLN/nodal outcomes. We excluded reviews, meta-analyses, conference abstracts, studies of endometrial cancer without a separable EIN/AEH subset, studies reporting only surgical technique, perioperative, or cost outcomes, without eligible concurrent carcinoma or nodal outcomes, and mixed cohorts in which the EIN/AEH subset could not be separated.

### 2.3. Data Extraction

Two reviewers (PCL and DCD) extracted the data independently. Data extraction captured study design, country, population terminology, denominator, concurrent-carcinoma events, unilateral and bilateral SLN detection, nodal positivity (macrometastasis, micrometastasis, and isolated tumor cells), changes in adjuvant treatment decisions, and complications.

#### Sensitivity Analysis

Because several included cohorts originated from the same country or research network, studies were cross-checked for overlapping patient populations by comparing enrolling institution(s), authorship, and recruitment period. Two candidate overlaps were identified: Capozzi et al. (2022, single-center, Italy) [10] and Rosati et al. (2024, multicenter, Italy) [3] share a co-author and may share source patients; and Matanes et al. (2023) [11] and Levin et al. (2024) [12], both single-institution Montreal cohorts of similar size, may likewise overlap. Enrollment dates could not be confirmed from the published texts, so independence could not be definitively established; sensitivity analyses excluding each candidate-overlap study were performed.

### 2.4. Quantitative Synthesis

Proportions were pooled using a random-effects (DerSimonian–Laird) meta-analysis on the Freeman–Tukey double-arcsine scale and back-transformed to percentages. Per-study Wilson 95% confidence intervals were displayed. Subgroup analyses pre-specified by histological terminology (EIN vs. AEH/atypical hyperplasia vs. mixed premalignancy) were performed for concurrent-carcinoma outcomes. Heterogeneity was quantified using I^2^. Analyses were performed using Python (version 3.14) with NumPy (version 2.5.2) and Matplotlib (version 3.11.1). The primary nodal-positivity outcome was defined as any pathologically confirmed tumor deposit (macrometastasis, micrometastasis, or isolated tumor cells) identified in a sentinel or regionally dissected node, applied uniformly across studies. A pre-specified sensitivity analysis excluded isolated tumor cells, given their distinct classification (pN0(i+)) and uncertain prognostic significance under FIGO/ISGyP conventions.

### 2.5. Risk of Bias Assessment

Risk of bias was assessed independently by two reviewers using the QUIPS (Quality in Prognosis Studies) tool across six domains [13]: study participation, study attrition, prognostic factor measurement, outcome measurement, study confounding, and statistical analysis and reporting. Each domain was rated as having a low, moderate, or high risk of bias, with disagreements resolved by consensus. QUIPS was selected, rather than a prevalence-specific tool, because the core relationship under evaluation, a preoperative diagnostic category (EIN/AEH, or subclassifications thereof) predicting a subsequent surgical/pathological outcome (concurrent carcinoma, nodal status), corresponds to a prognostic factor rather than a purely descriptive-prevalence question, and because QUIPS uniquely incorporates a confounding domain capable of capturing the surgeon-driven, indication-based selection of patients for SLN mapping that we identified as a major source of bias (Section 3.5). Each domain was rated as having a low, moderate, or high risk of bias, with disagreements resolved by consensus.

## 3. Results

### 3.1. Study Selection

The database searches identified 153 records (PubMed, *n* = 55; Embase, *n* = 97; and Cochrane, *n* = 1). After removal of 74 duplicates, 79 records underwent title and abstract screening; 21 were excluded, and 58 reports underwent full-text assessment. Forty reports were excluded (eight reviews/meta-analyses, 13 conference abstracts, 10 endometrial-cancer-only cohorts without a separable EIN/AEH subset, eight studies reporting only technique or perioperative outcomes, and one mixed cohort in which the EIN/AEH subset could not be separated), leaving 18 studies identified through the database searches. One additional study [14] was identified through supplementary searching, yielding 19 included studies (Table 1, Figure 1). The studies were published between 2018 and 2026, represented nine countries, and were all retrospective. Across the 19 studies, 13,553 women with a preoperative diagnosis of EIN/AEH were included; after exclusion of the large administrative-database cohort (Dioun 2021 [7], n = 10,266), the remaining 18 studies included 3287 patients, and 14 studies comprising 1174 patients contributed to the pooled nodal-positivity estimate.

### 3.2. Concurrent Endometrial Carcinoma

Eighteen studies reported concurrent endometrial carcinoma on final hysterectomy pathology (Figure 2). The pooled rate was 39.6% (95% CI 34.9–44.3; I^2^ = 86%). By diagnostic terminology, the pooled rate was 43.8% (95% CI 39.7–48.0; I^2^ = 54%) in AEH/atypical-hyperplasia cohorts and 33.4% (95% CI 28.1–39.0; I^2^ = 79%) in EIN cohorts; the rate in the single mixed-premalignancy cohort was 44.8%. Heterogeneity was high.

### 3.3. Nodal Positivity

Fourteen studies reported nodal positivity among women undergoing SLN mapping or other nodal assessment (Figure 3). The pooled positivity rate was 3.0% (95% CI 2.1–4.0; I^2^ = 0%). In a sensitivity analysis excluding Sullivan et al. [5]—in which only three of the seven nodal-assessment patients underwent sentinel lymph node biopsy, the remaining four underwent full pelvic lymphadenectomy, with 0/7 nodal metastases overall—the pooled estimate remained essentially unchanged at 2.8% (95% CI 1.9–3.8; I^2^ = 0%), indicating that inclusion of this mixed-nodal-assessment cohort did not materially bias the primary SLN-focused nodal-positivity estimate.

In a second sensitivity analysis restricted to macrometastases and micrometastases only (excluding isolated tumor cells), the pooled positivity rate was 2.6% (95% CI, 1.7–3.7; I^2^ = 15%), compared with 3.0% (95% CI, 2.1–4.0; I^2^ = 0%) when isolated tumor cells were included as events.

### 3.4. Detection Rate, Safety, and Adjuvant Impact

The pooled bilateral SLN detection rate was 71.6% (95% CI 67.3–75.7; I^2^ = 0%) (Figure 4), while unilateral detection rates were approximately 87–95% across studies. SLN mapping was not associated with excess morbidity. SLN findings influenced adjuvant therapy decisions in approximately 8–15% of all SLN-mapped patients across studies [11,17,20,21]; this proportion rises substantially when calculated among narrower denominators. In the most recently added cohort, SLN status modified treatment decisions in 12.3% of all SLN-mapped high-to-intermediate-risk patients but in 11 of 22 (50%) patients who ultimately received adjuvant therapy [14], illustrating that the clinical impact of SLN findings is denominator-dependent and greatest among patients for whom adjuvant treatment is already being considered. No recurrences were reported in several studies during the available follow-up periods [2,5].

Sensitivity analyses excluding the candidate-overlap cohorts (Capozzi 2022 [10] alone, Levin 2024 [12] alone, or both) changed the pooled concurrent-carcinoma rate by less than 0.5 percentage points (39.6–40.1%) (Table 2). The pooled nodal-positivity rate was negligible (3.0% vs. 2.85% excluding Capozzi 2022 [10]) (Table 3). The above indicates that the primary estimates are robust to possible cohort overlap.

### 3.5. Risk of Bias

All 19 studies had an overall moderate risk of bias (Figure 5). Outcome measurements (pathology and SLN status) were generally at low risk of bias, whereas concerns regarding confounding and selection bias were moderate. Across the six QUIPS domains, moderate ratings were driven primarily by study confounding and study participation: SLN mapping was typically performed at the operating surgeon’s discretion in patients selected for higher-risk preoperative features (e.g., thickened endometrial stripe, elevated CA-125, or “cannot exclude carcinoma” histology), introducing indication bias, compounded by retrospective, non-consecutive enrollment across single- and multicenter cohorts that raised concerns about representativeness. In contrast, outcome measurement (final pathology and SLN status) and study attrition were generally rated low risk, as these were objectively and near-completely ascertained in most cohorts, while prognostic factor measurement and statistical analysis and reporting were rated low to moderate.

## 4. Discussion

This updated synthesis of 19 studies yielded two principal findings. First, concurrent endometrial carcinoma was common, occurring in approximately 40% of women with preoperative EIN/AEH; the pooled rate was higher in cohorts labeled atypical hyperplasia (44%) than in those labeled as EIN (33%). Individual estimates ranged from 23.8% [9] to 55% [16]. Among studies reporting tumor characteristics, concurrent cancers were predominantly low-grade, early-stage endometrioid tumors (e.g., 96–100% endometrioid, with most classified as FIGO stage IA) [11,17,24]. Heterogeneity in the concurrent-carcinoma estimate was substantial (I^2^ = 86%) and may reflect differences in diagnostic sampling, referral patterns, and case definitions. This heterogeneity likely also reflects coexisting diagnostic frameworks: the older WHO 1994 “complex atypical hyperplasia” criterion is pattern-based and subject to substantial interobserver variability, whereas EIN uses more reproducible, morphometry-derived criteria intended to capture a molecularly defined clonal lesion [25]. The WHO 2014/2020 schemes treat “AH/EIN” as interchangeable without fully harmonizing thresholds [26,27]. Since most studies relied on local rather than centralized pathology review, terminology-linked diagnostic drift may partly explain the higher concurrent-carcinoma rate in AEH/CAH-labeled cohorts (43.8%) versus EIN-labeled cohorts (33.4%), and the subgroup estimates should be read as approximate rather than proof of a true biological difference. Second, despite the high burden of occult carcinoma, nodal metastasis was uncommon (3.0%) and, when present, was generally low-volume. No statistical heterogeneity was detected in the pooled nodal-positivity estimate (I^2^ = 0%), although the low frequency of nodal events should be considered when interpreting this finding. The clinical significance of such low-volume disease is itself unsettled: a meta-analysis of endometrial cancer patients with sentinel-node micrometastases or isolated tumor cells reported a modestly increased recurrence risk relative to node-negative disease (RR 1.34, 95% CI 1.07–1.67) even with adjuvant therapy [28], whereas other series have found near-negligible risk among low-grade, lymphovascular-invasion-negative tumors. Because this evidence derives almost entirely from general endometrial carcinoma cohorts rather than patients with a preoperative diagnosis of EIN/AEH specifically, the prognostic and therapeutic implications of low-volume nodal disease in this population remain uncertain.

These findings frame the clinical question regarding routine SLN mapping in EIN/AEH. The argument for routine SLN mapping rests on the high concurrent cancer rate [11], while the argument against routine use rests on the low nodal-positivity rate and the fact that most positive nodes are micrometastases or isolated tumor cells of uncertain therapeutic significance [2]. The most recently added cohort [14] reported SLN metastases in four of 109 SLN-assessed patients with occult carcinoma (3.7%). Across studies, isolated tumor cells and micrometastases outnumbered macrometastases, and several cohorts reported no nodal metastases [5,16,20]. The highest individual estimate, 7.6% [3], was calculated only among patients with concurrent carcinoma undergoing nodal assessment rather than the entire EIN/AEH cohort. Our data support the latter: based on the pooled nodal-positivity rate of 3.0%, approximately 33 women who underwent nodal assessment would need to be mapped to identify one node-positive patient. Because SLN mapping was performed selectively, typically in patients with higher-risk preoperative features (Section 3.5), this figure applies to an already risk-enriched subgroup rather than an unselected EIN/AEH population; the corresponding number needed to map in an unselected cohort, where lower-risk patients would presumably dilute the nodal-positivity rate further, is likely higher still. Most concurrent cancers were nonetheless low-grade and uterine-confined, for which nodal status would be unlikely to change management. This finding is consistent with the conclusion of the 2024 meta-analysis [8] but is based on a larger evidence base with a tighter confidence interval.

The potential clinical value of SLN mapping should therefore be concentrated in selected patients [3]. The pooled bilateral detection rate of approximately 72% indicates that SLN mapping is technically feasible. Unilateral detection rates of approximately 87–95% across studies were comparable to those reported in endometrial cancer SLN series. Studies reporting comparative complication outcomes found no excess morbidity with SLN mapping compared with that via hysterectomy alone [2,17,21]. An administrative-database cohort excluded from quantitative synthesis for the reasons noted above also reported a lower complication rate with SLN mapping than with lymphadenectomy (5.2% vs. 6.9%) [7]; given its reliance on billing codes rather than adjudicated pathology or complication data, this observation is noted only as hypothesis-generating and was not used to support any conclusion of this review.

SLN findings influenced adjuvant treatment decisions in approximately one in eight patients, particularly among those upstaged to intermediate- or high-risk carcinoma [3,11,17,21]. Several studies also reported no recurrences during the available follow-up period [2,5], although the generally short follow-up limits conclusions regarding longer-term oncologic outcomes. A risk-stratified approach (Table 4), reserving SLN for patients whose preoperative features (older age, thickened endometrial stripe, diagnosis by biopsy rather than curettage, obesity, hyperlipidemia, bleeding pattern, elevated CA-125, or “cannot exclude carcinoma” histology) predict higher-risk concurrent cancer, captures most of the benefit while sparing the majority an unnecessary procedure [12,18,19,23].

Prospective validation of this selective approach has since emerged. Peters et al. reported a preoperative risk-stratification algorithm in which an endometrial stripe ≥15 mm and/or a pathology report that could not exclude carcinoma triggered an indocyanine green SLN biopsy, whereas patients below this threshold underwent hysterectomy alone [29]. Among 50 women with EIN, the algorithm achieved 92% sensitivity (22/24) for surgical staging of patients with cancer and 100% sensitivity (8/8) for those with high-risk cancer, at a specificity of 63% and a negative predictive value of 89%. These prospective findings directly corroborate the present pooled analysis: rather than routine SLN mapping for all women with EIN/AEH, a preoperative risk-stratified algorithm can capture nearly all clinically significant disease while sparing a substantial proportion of low-risk patients an unnecessary nodal procedure.

Our findings can also be contextualized in relation to two recent meta-analyses on this topic. Nahshon et al. [8] pooled seven studies using any form of nodal assessment (SLN or lymphadenectomy) and reported a positive-node rate of 1.1%. The present analysis similarly demonstrated a low nodal-positivity rate, although the pooled estimate was numerically higher at 3.0% and was based on a larger evidence base. Vieira-Serna et al. [30] restricted their meta-analysis to studies reporting SLN-specific outcomes and included only four studies, three of which [2,11,18] were also included in the present review. They reported an SLN positivity rate of 1.6% among patients with preoperative EIN/AEH and 3.5% among those with concurrent carcinoma, together with a concurrent carcinoma rate of 45%, which is broadly consistent with the present findings. By including studies that used both SLN mapping and other forms of nodal assessment, our meta-analysis incorporated a broader range of nodal evaluation strategies. Vieira-Serna et al. concluded that routine SLN evaluation may not be necessary in EIN/AEH given the low positivity rate [30]. Nevertheless, this low yield should be weighed against the potential of nodal status to inform adjuvant treatment decisions in selected patients, particularly given the lower procedural burden of SLN mapping compared with full lymphadenectomy.

A specific circularity warrants comment. The pooled nodal-positivity estimate was calculated among patients who underwent nodal assessment, a group already informally enriched for higher-risk preoperative features by treating surgeons (Section 3.5). Consequently, a low yield in this already-selected group does not, on its own, demonstrate that formal risk-based selection criteria would concentrate nodal-positive patients more efficiently than routine assessment; it is equally consistent with the possibility that currently used clinical risk features have limited discriminatory power, and that the true nodal-positivity rate in a fully unselected EIN/AEH population is lower still. This distinguishes our data from a genuine test of a selective strategy, which requires prospective application of predefined criteria to an unselected cohort—an approach recently piloted on a small scale by Peters et al. [29]. We therefore present our support for a selective over a routine approach as hypothesis-generating rather than as a directive recommendation, pending validation of specific selection criteria in larger prospective cohorts.

The study has a few limitations. All included studies were retrospective and had a moderate risk of bias, with the selection of higher-risk patients for SLN mapping potentially increasing the observed yields. Heterogeneity in the pooled concurrent-carcinoma estimate was high; therefore, it should be interpreted as a broad summary across heterogeneous study populations rather than as a universally applicable value. Terminology (EIN, AEH, and complex atypical hyperplasia) and nodal-assessment denominators varied across studies; terminology was examined in subgroup analyses, while nodal positivity was pooled across patients undergoing nodal assessment. Prediction and administrative-database studies contributed only to selected outcomes. Finally, the follow-up period was generally short, limiting inferences regarding recurrence and survival. Because the deposit type (ITC vs. micrometastasis vs. macrometastasis) was not uniformly reported across studies and carries different prognostic and therapeutic significance under FIGO/ISGyP conventions, the pooled nodal-positivity rate should be interpreted as a composite estimate rather than one specific to a single metastasis category.

Prospective, risk-stratified evaluations of SLN mapping in EIN/AEH, standardized reporting of EIN/AEH-specific denominator, and analyses of the prognostic significance of isolated tumor cells and micrometastases in this population would help clarify the role of SLN assessment in clinical practice.

## 5. Conclusions

In women with a preoperative diagnosis of EIN/AEH, concurrent endometrial carcinoma is common (approximately 40%); however, nodal metastasis is uniformly rare (3.0%) and usually low-volume. SLN mapping appears feasible, with no clear excess in reported complications, and may inform adjuvant treatment decisions in a minority of patients. However, because the low nodal yield was observed largely among patients already selected for higher-risk features rather than in an unselected population, this finding should be interpreted as hypothesis-generating rather than definitive support for a selective, risk-stratified approach; such an approach remains plausible and is preliminarily supported by early prospective data, but requires validation of specific selection criteria in larger prospective cohorts before it can be recommended with confidence.

## Figures and Tables

**Figure 1:**
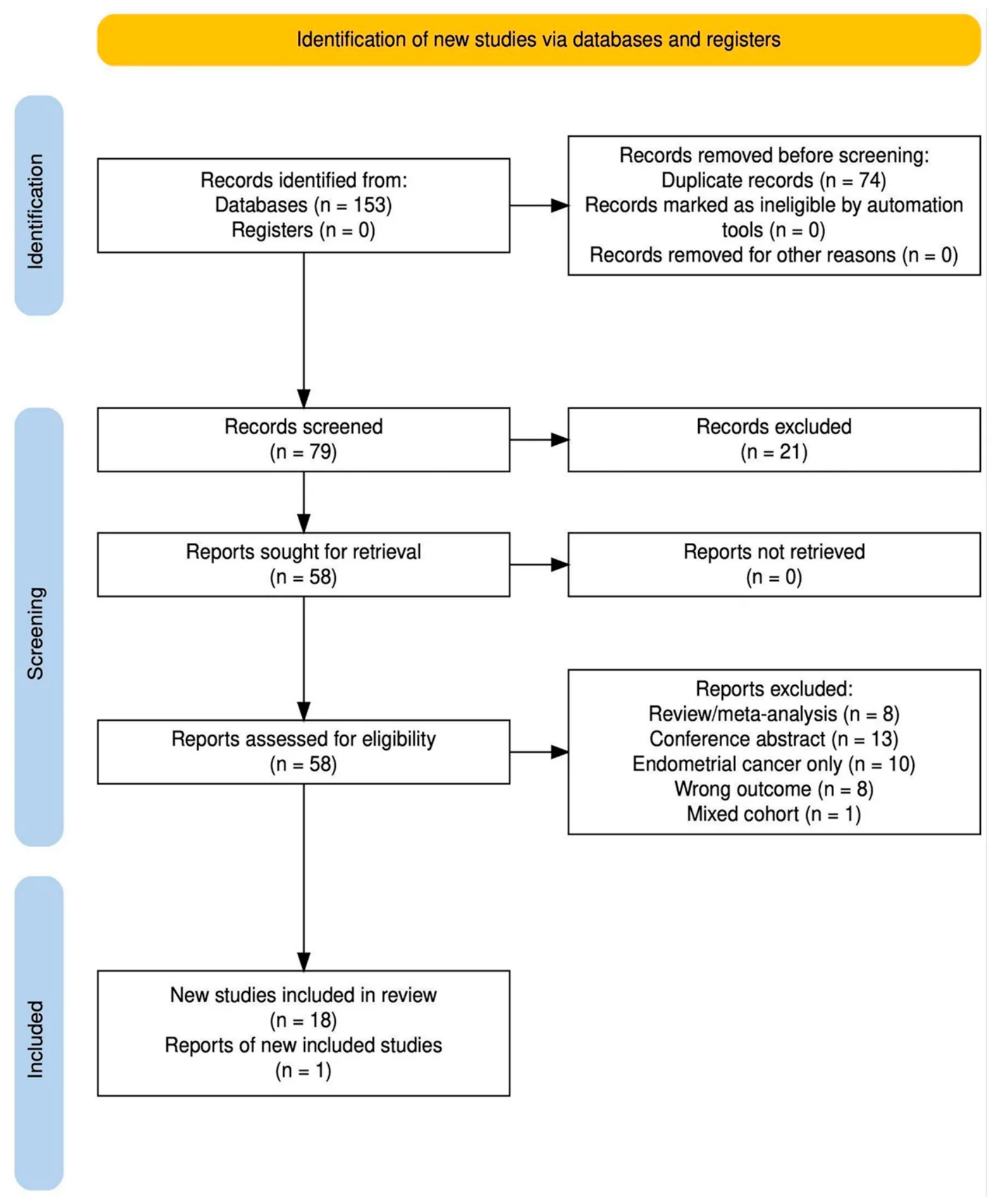
PRISMA 2020 flow diagram (153 database records + 1 via further searching, yielding 19 included studies).

**Figure 2:**
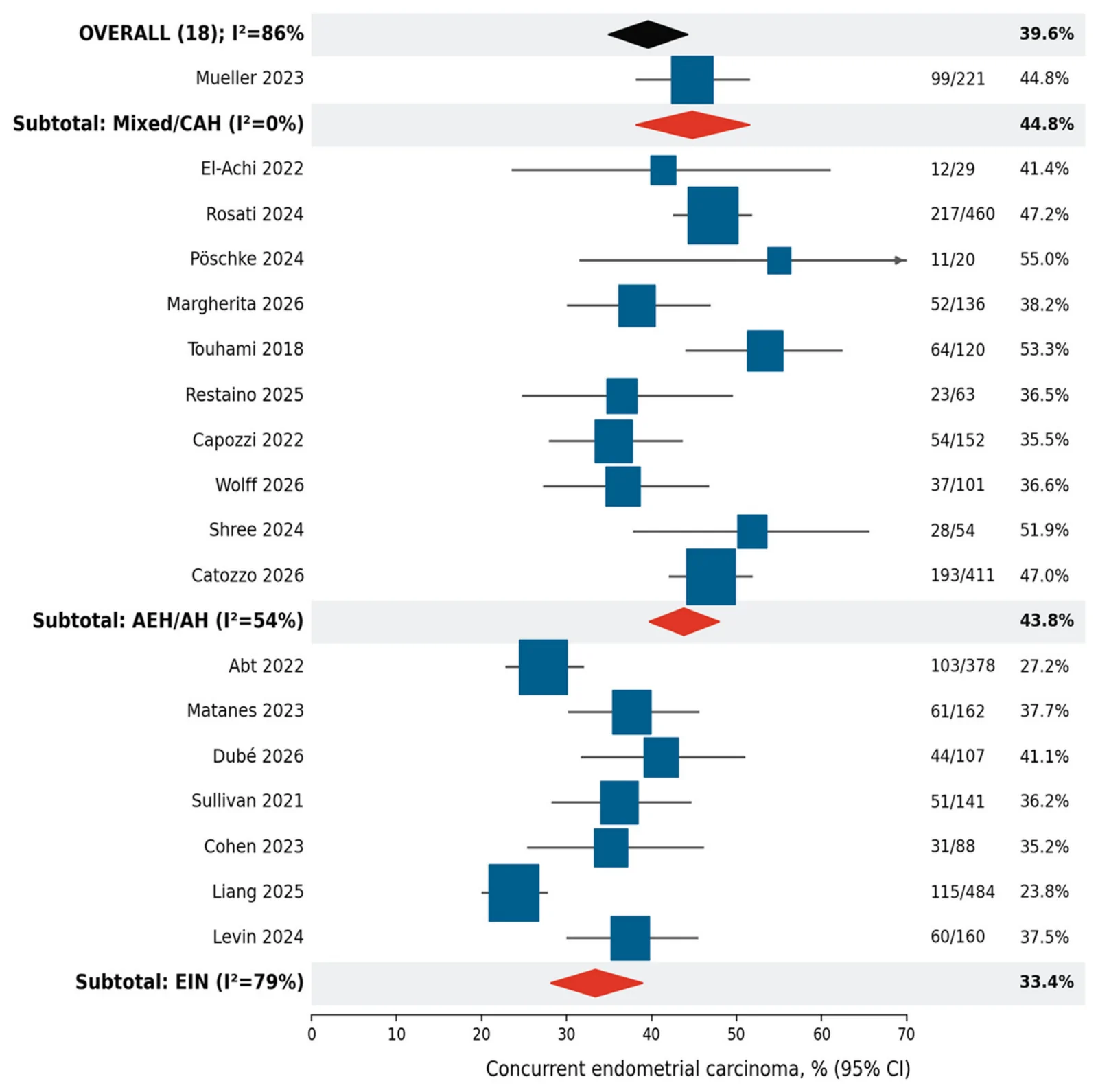
Concurrent endometrial carcinoma in preoperative EIN/AEH, with terminology subgroups [2,3,9,10,11,12,14,15,16,17,18,19,20,21,22,23,24]. Pooled 39.6% (95% CI 34.9–44.3). Black diamond: overall meta-analysis. Red diamond: subgroup meta-analysis. Square: percentage in the study.

**Figure 3:**
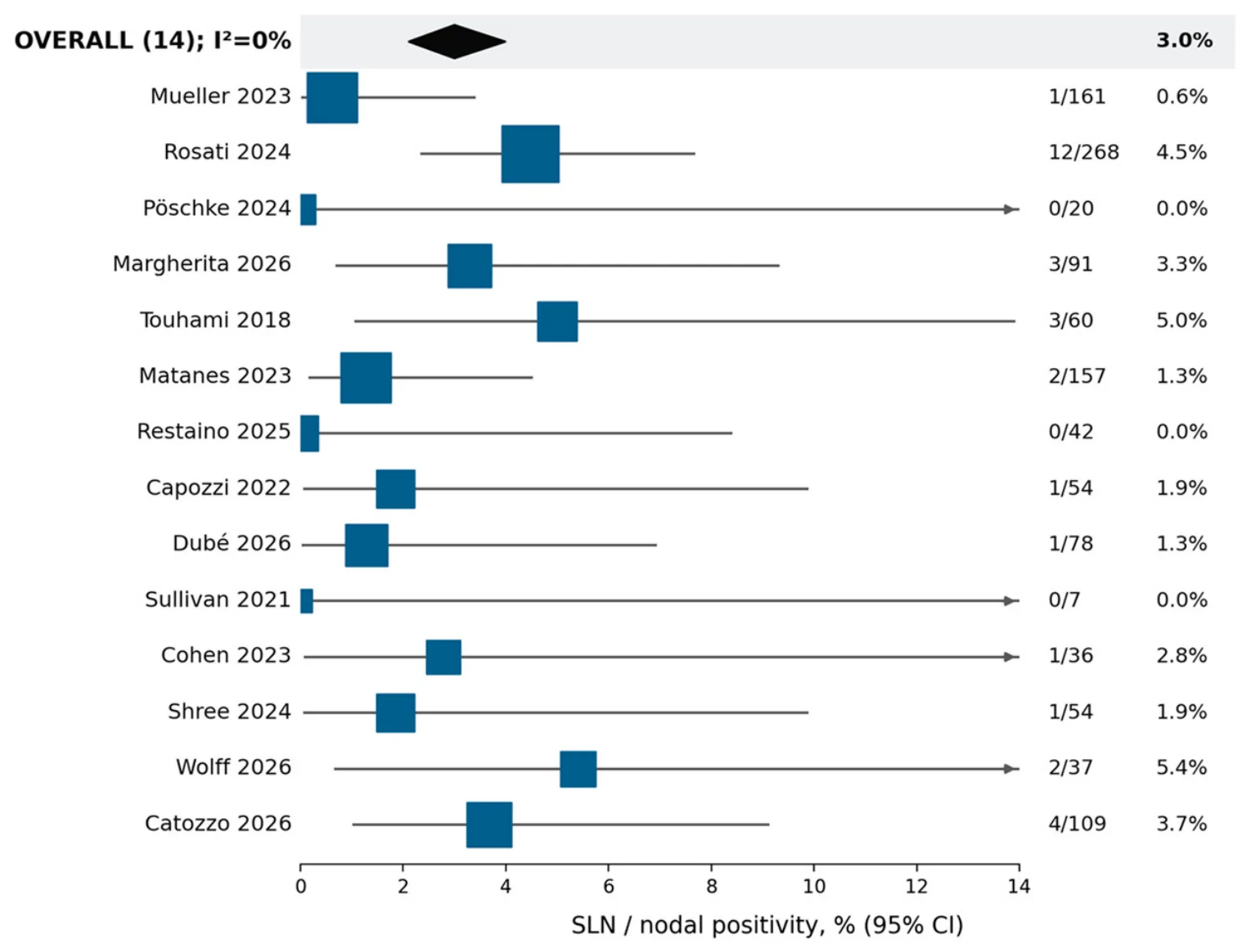
Sentinel lymph node/nodal positivity. Pooled 3.0% (95% CI 2.1–4.0; I^2^= 0%) [2,3,5,10,11,14,16,17,18,20,21,22,24]. Black diamond: overall meta-analysis. Square: percentage in the study.

**Figure 4:**
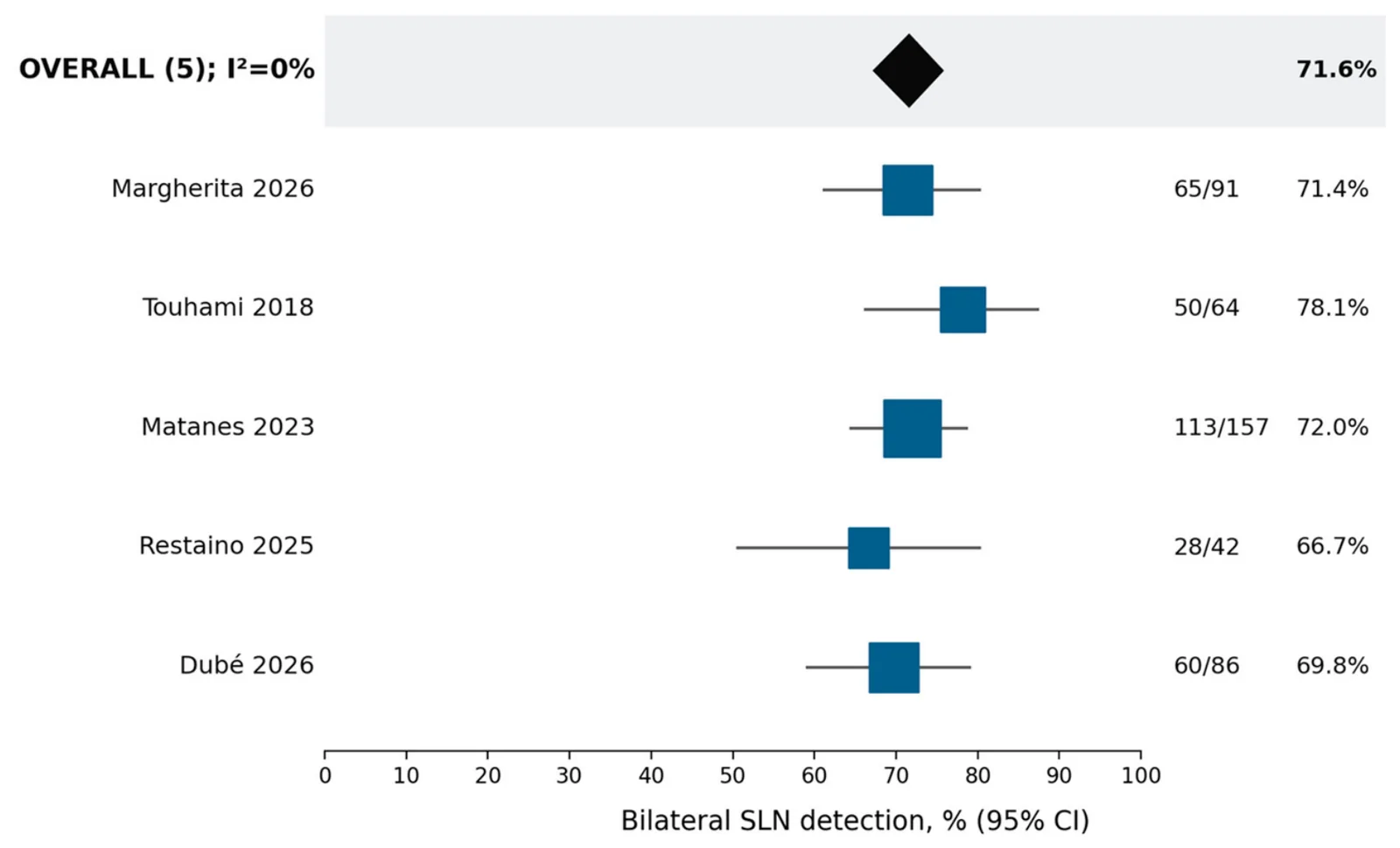
Bilateral SLN detection rate. Pooled 71.6% (95% CI 67.3–75.7) [11,17,18,20,21]. Black diamond: overall meta-analysis. Square: percentage in the study.

**Figure 5:**
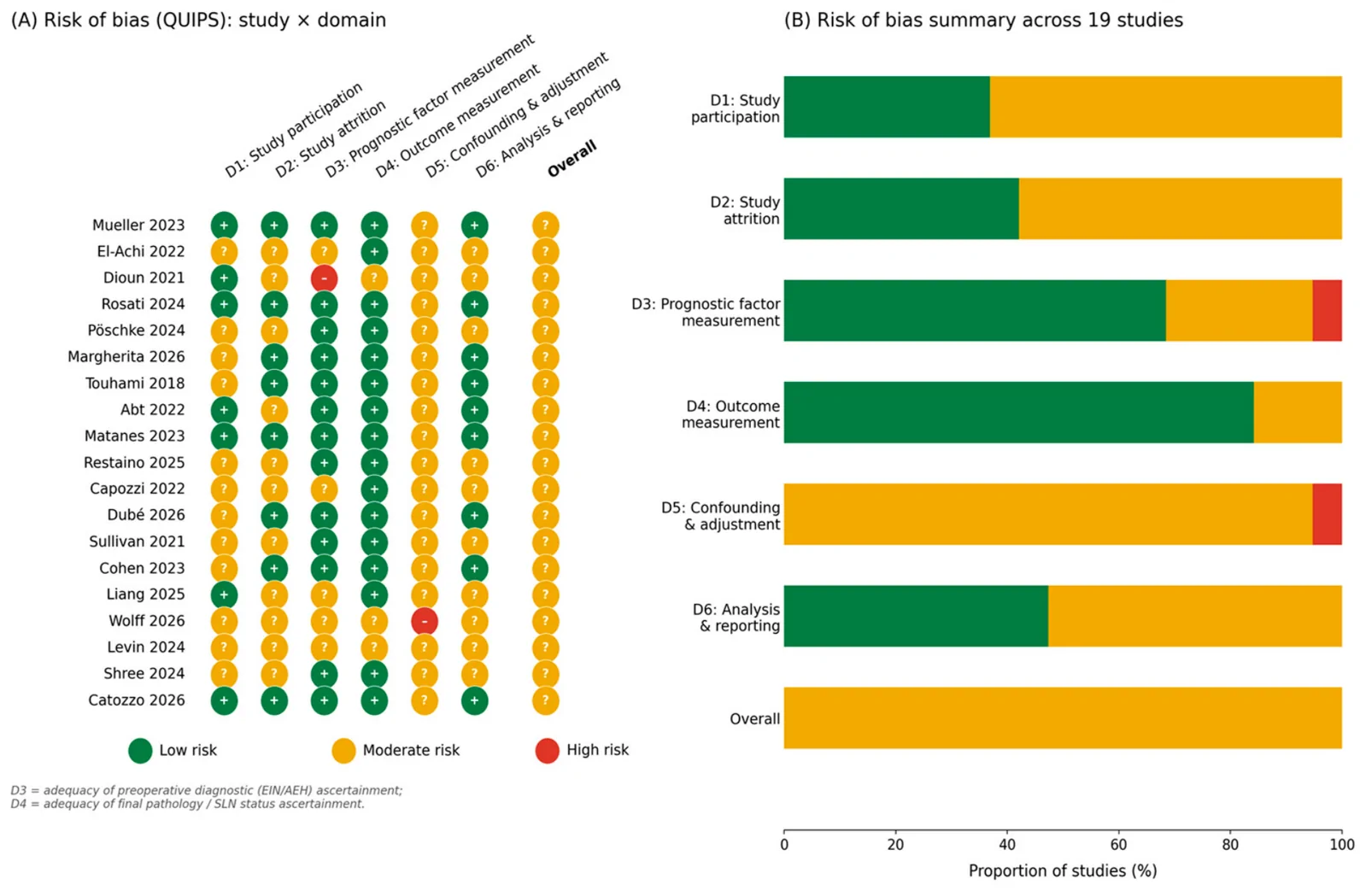
Risk of bias (QUIPS) across the 19 included studies [2,3,5,7,9,10,11,12,14,15,16,17,18,19,20,21,22,23,24].

**Table 1:** Characteristics and outcomes of the 19 included studies.

#	Study	Year	Country	Design	Population	N	Concurrent EC	Nodal Positivity
1	Mueller et al. [2]	2023	USA	Retrospective	EIN/CAH/AH	221	99/221 (45%)	1/161 +1 ITC
2	El-Achi et al. [15]	2022	Australia	Retrospective	AH (+EC)	~29	12/29 (42%)	n/r (AH)
3	Dioun et al. [7]	2021	USA	Admin database	CAH	10,266	NA (billing)	NA
4	Rosati et al. [3]	2024	Italy	Retrospective multicenter	AEH	460	217/460 (47%)	12/268 (4.5%)
5	Pöschke et al. [16]	2024	Germany	Retrospective	AEH	20	11/20 (55%)	0/20
6	Margherita et al. [17]	2026	Italy	Retrospective	AEH	136	52/136 (38%)	3/91 (3.3%)
7	Touhami et al. [18]	2018	Canada	Retrospective	AH	120	64/120 (53%)	3/60 (5%)
8	Abt et al. [19]	2022	USA	Retrospective	EIN	378	103/378 (27%)	limited eval
9	Matanes et al. [11]	2023	Canada	Retrospective	EIN	162	61/162 (38%)	2/157 (1.2%)
10	Restaino et al. [20]	2025	Italy	Retrospective	AH	63	23/63 (37%)	0/42
11	Capozzi et al. [10]	2022	Italy	Retrospective	AH	152	54/152 (36%)	1/54 (0.7%)
12	Dubé et al. [21]	2026	Canada	Retrospective 2-center	EIN	107	44/107 (41%)	1/78 (1.2%)
13	Sullivan et al. [5]	2021	USA	Retrospective	AH/EIN	141	51/141 (36%)	0/7
14	Cohen et al. [22]	2023	Israel	Retrospective	EIN (P/E)	88	31/88 (35%)	1/36 (2.8%)
15	Liang et al. [9]	2025	China	Retrospective nomogram	EIN	484	115/484 (24%)	—
16	Wolff et al. [23]	2026	Israel	Retrospective case–control	AEH	101	37/101 (37%)	2/37 (5.4%)
17	Levin et al. [12]	2024	Canada	Retrospective ML	EIN	160	60/160 (38%)	—
18	Shree et al. [24]	2024	India	Retrospective	AEH	54	28/54 (52%)	1/54 (1.8%)
19	Catozzo et al. [14]	2026	Italy	Retrospective multicenter	AEH/EIN	411	193/411 (47%)	4/109 (3.7%)

ITC: Isolated Tumor Cells.

**Table 2:** Concurrent endometrial carcinoma (original analysis: 39.6%, 95% CI 34.9–44.3; I^2^ = 86%; k = 18).

Exclusion	Pooled Rate	95% CI	I^2^
Excl. Capozzi 2022 [10]	39.9%	35.0–44.9	87%
Excl. Levin 2024 [12]	39.8%	34.8–44.8	87%
Excl. both	40.1%	34.8–45.4	87%

Excl: excluding. CI: confidence interval.

**Table 3:** Nodal/SLN positivity (original analysis: 3.0%, 95% CI 2.1–4.0; I^2^ = 0%; k = 14).

Exclusion	Pooled Rate	95% CI	I^2^
Excl. Capozzi 2022 [10]	2.85%	1.9–4.0	5%

Excl: excluding. CI: confidence interval.

**Table 4:** Preoperative features reported to predict higher-risk concurrent carcinoma in EIN/AEH.

Preoperative Feature	Reported Association	Source
Endometrial strip ≥20 mm on ultrasound	RR 1.97 (95% CI 1.34–2.90) for concurrent carcinoma	Abt et al. [19]
Diagnosis by office/pipelle biopsy vs. hysteroscopy/D& C	OR 3.80 (95% CI 1.21–11.93)	Wolff et al. [23]
Obesity	OR 3.33 (95% CI 1.14–9.73)	Wolff et al. [23]
Hyperlipidemia	OR 3.95 (95% CI 1.40–11.09)	Wolff et al. [23]
Age	OR 1.07 per year (95% CI 1.01–1.14)	Wolff et al. [23]
PMB vs. AUB	OR 3.95 (95% CI 1.40–11.09)	Wolff et al. [23]
“Cannot exclude carcinoma” on preoperative histology	Nodal metastasis in 12.1% (4/33) of cancer cases vs. 0% (0/31) with “AH-only” histology	Touhami et al. [18]
CA-125, endometrial thickness (as model inputs, not independently validated)	Included as candidate predictors in a multivariable ML model	Levin et al. [12]

RR: relative risk, OR: odds ratio, CI: confidence interval, D & C: dilation and curettage, AH: atypical hyperplasia, ML: machine learning, EIN/AEH: endometrial intraepithelial neoplasia/atypical endometrial hyperplasia, AUB: abnormal uterine bleeding, PMB: postmenopausal bleeding.

## Data Availability

No new data were created or analyzed in this study. Data sharing is not applicable to this article.

## Supplementary Materials

The following supporting information can be downloaded at: https://www.mdpi.com/article/10.3390/diagnostics16193114/s1. File S1: Prisma 2020 Checklist; File S2: Search strategies. Reference [31] is cited in the supplementary materials.

## Abbreviations

The following abbreviations are used in this manuscript:

EIN	Endometrial intraepithelial neoplasia
AEH	Atypical endometrial hyperplasia
AH	Atypical hyperplasia
SLN	Sentinel lymph node
OR	Odds ratio
HR	Hazard ratio
AUB	Abnormal uterine bleeding
PMB	Postmenopausal bleeding
ML	Machine learning
CI	Confidence interval
D & C	Dilation and curettage
RR	Relative risk
WHO	World Health Organization
FIGO	International Federation of Gynecology and Obstetrics
ITC	Isolated Tumor Cells
QUIPS	Quality in prognosis studies
