# Supplementary material for: Sentinel Lymph Node Assessment in Endometrial Intraepithelial Neoplasia/Atypical Endometrial Hyperplasia: An Updated Systematic Review and Meta-Analysis
Source: Diagnostics (Basel). 2026 Sep 25;16(19):3114. doi: 10.3390/diagnostics16193114 (PMC13664625; doi:10.3390/diagnostics16193114)
Supplement: Supplementary file 1 [file diagnostics-16-03114-s001.zip › File S2.pdf]

Supplementary Materials

Search strategies: Two concepts combined with AND: (1) EIN / atypical endometrial hyperplasia AND (2) sentinel lymph node / nodal assessment. Searches run through 2026; date de-duplicated and screened in Rayyan. Yields: PubMed 55, Embase 97, Cochrane CENTRAL 1 (153 total; 74 duplicates removed; 79 screened).

PubMed

#1 ("Endometrial Hyperplasia"[Mesh] OR "endometrial hyperplasia"[tiab]
OR "endometrial intraepithelial neoplasia"[tiab] OR EIN[tiab]
OR "atypical hyperplasia"[tiab] OR "atypical endometrial hyperplasia"[tiab]
OR "complex atypical hyperplasia"[tiab] OR AEH[tiab])
#2 ("Sentinel Lymph Node"[Mesh] OR "Sentinel Lymph Node Biopsy"[Mesh]
OR "Lymph Node Excision"[Mesh]
OR "sentinel lymph node*"[tiab] OR "sentinel node*"[tiab] OR SLN[tiab]
OR "lymph node mapping"[tiab] OR "nodal assessment"[tiab]
OR "lymphadenectomy"[tiab] OR "lymph node dissection"[tiab] OR "lymph node biopsy"[tiab])
#3 #1 AND #2

Embase (Embase.com)

#1 ('endometrium hyperplasia'/exp OR 'endometrial intraepithelial neoplasia':ab,ti
OR ein:ab,ti OR 'endometrial hyperplasia':ab,ti OR 'atypical hyperplasia':ab,ti
OR 'atypical endometrial hyperplasia':ab,ti OR 'complex atypical hyperplasia':ab,ti OR aeh:ab,ti)
#2 ('sentinel lymph node'/exp OR 'sentinel lymph node biopsy'/exp OR 'lymph node dissection'/exp
OR 'sentinel lymph node':ab,ti OR 'sentinel node':ab,ti OR sln:ab,ti
OR 'lymph node mapping':ab,ti OR 'nodal assessment':ab,ti OR lymphadenectomy:ab,ti OR 'lymph node biopsy':ab,ti)
#3 #1 AND #2

Cochrane CENTRAL (Search Manager)

#1 MeSH descriptor: [Endometrial Hyperplasia] explode all trees
#2 ("endometrial hyperplasia" OR "endometrial intraepithelial neoplasia" OR EIN
OR "atypical hyperplasia" OR "atypical endometrial hyperplasia"
OR "complex atypical hyperplasia" OR AEH):ti,ab,kw
#3 #1 OR #2
#4 MeSH descriptor: [Sentinel Lymph Node] explode all trees
#5 MeSH descriptor: [Sentinel Lymph Node Biopsy] explode all trees
#6 MeSH descriptor: [Lymph Node Excision] explode all trees
#7 ("sentinel lymph node*" OR "sentinel node*" OR SLN OR "lymph node mapping"
OR "nodal assessment" OR lymphadenectomy OR "lymph node dissection" OR "lymph node biopsy"):ti,ab,kw
#8 #4 OR #5 OR #6 OR #7
#9 #3 AND #8
